# The “outsized” role of the I‐helix kink in human Cytochrome P450s

**DOI:** 10.1002/ctm2.1378

**Published:** 2023-09-15

**Authors:** Jingjing Zhang, Fengting Liu, Yaran Suo, Dudu Tong, Jinyu Hu, Hai‐Ning Lyu, Jingjing Liao, Jiaqi Wang, Jigang Wang, Chengchao Xu

**Affiliations:** ^1^ Department of Nephrology Shenzhen Key Laboratory of Kidney Diseases Shenzhen Clinical Research Centre for Geriatrics Shenzhen People's Hospital, The First Affiliated Hospital Southern University of Science and Technology Shenzhen P. R. China; ^2^ Integrated Chinese and Western Medicine Postdoctoral Research Station Jinan University Guangzhou P. R. China; ^3^ State Key Laboratory for Quality Ensurance and Sustainable Use of Dao‐di Herbs, Artemisinin Research Center and Institute of Chinese Materia Medica China Academy of Chinese Medical Sciences Beijing P. R. China; ^4^ School of Pharmaceutical Sciences (Shenzhen) Shenzhen Campus of Sun Yat‐sen University Shenzhen P. R. China; ^5^ College of Integrative Medicine Laboratory of Pathophysiology Key Laboratory of Integrative Medicine on Chronic Diseases Fujian University of Traditional Chinese Medicine Fuzhou P. R. China

Dear Editor,

Cytochrome P450s (CYPs) are a superfamily of heme‐containing enzymes that play critical roles in oxidizing endogenous metabolites and xenobiotics.^1^ They are membrane‐anchored enzymes, and the transmembrane domains are involved in the electron transfer and the access of substrate and water during the catalytic cycle.^2^, ^3^, ^4^ Mutations of human CYPs cause metabolic disorders and abnormal drug metabolism.^5^ Because their amino acid sequences are remarkably diverse, the existing work on different human CYPs is seemingly unrelated. However, it is important to note that the overall structural folds of CYPs are largely conserved.^6^ We, therefore, wondered if there is any shared feature of functionally unrelated human CYPs that is linked to enzyme malfunction and human disorders.

The oxygen‐binding motif is relatively conserved among different CPYs (Figure 1A), which locates in the central region of the I‐helix and is implicated in the delivery of protons from water molecules to bound dioxygen during the catalytic cycle.^6^, ^7^, ^8^, ^9^ Interestingly, a closer examination of a few unrelated CYP structures revealed that the first two amino acids of the oxygen‐binding motif produce a kink in the I‐helix^6^ (Figure 1B). Previous studies showed that mutations of this kink in CYP21A2 (G292S/C/R/D) and CYP11B1 (G314R) disrupt the biosynthesis of cortisol and aldosterone and lead to inherited congenital adrenal hyperplasia.^10^, ^11^ Because the kink is in close proximity with the oxidized moiety of the substrate and the heme iron, we hypothesized that the amino acid composition might be crucial to shaping the enzyme pocket environment, and changes could have a profound impact on enzyme activity for all CYPs. Interestingly, we found 84 non‐synonymous single‐nucleotide polymorphisms (SNPs) in this kink region of human CYPs from the NCBI database of genetic variation (dbSNP), most of which remained uncharacterized (coloured in blue, Figure 1C; Table S1). Among them, only four, including G292R/S of CYP21A2,^10^ G314R of CYP11B1^11^ and G312R of CYP2J2,^12^ have been characterized, which greatly diminish the monooxygenation activities.

**FIGURE 1:**
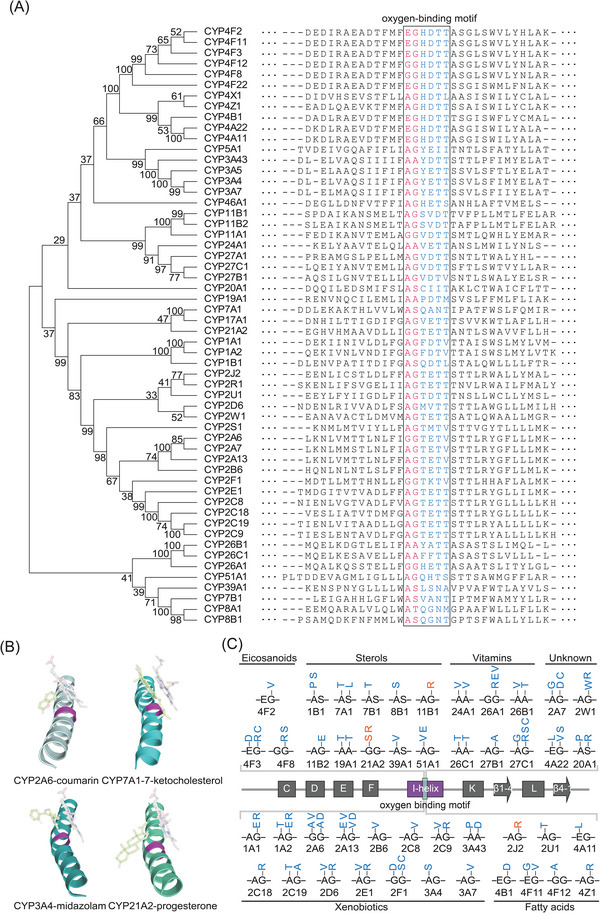
The I‐helix kink and non‐synonymous single‐nucleotide polymorphisms (SNPs) in human Cytochrome P450s (CYPs). (A) The phylogenetic tree of different human CYPs and the alignment of the I‐helix sequence. The oxygen‐binding motifs are shown in the rectangle box. The two amino acids of the I‐helix kink are highlighted in red. (B) The feature of the I‐helix kink (coloured in magenta) in four unrelated CYPs, including the human CYP2A6‐coumarin (pale cyan, PDB: 1Z10), CYP7A1‐7‐ketocholseterol (cyan, PDB: 3V8D), CYP3A4‐midazolam (deep steel, PDB: 5TE8), and CYP21A2‐progesterone (green, PDB: 4Y8W) complexes. In each structure, the heme iron is shown in light grey, and the corresponding substrate is highlighted in light green. (C) A summary of the non‐synonymous SNPs at the I‐helix kink in 45 human CYPs. The characterized SNPs are labelled in orange, and the uncharacterized ones are in blue. The grey rectangles represent the domain that compromises the core structure of mammalian CYPs, including helix C, D, E, F, I, K, L and two β strands.

To thoroughly analyze how the changes of the kink might affect the activity of CYPs in general, we selected three functionally unrelated CYPs—CYP21A2, CYP7A1 and CYP2A6, as an example. CYP21A2 is involved in hormone metabolism and oxidizes progesterone to 21‐OH progesterone. We transformed the wild‐type (WT) CYP21A2 and the mutants of the I‐helix kink (G292A, G292S/SNP, G292V, G292R/SNP, G293A, G293S and G293V) into 293T/17 cells (Figure S1), and fed the cells with progesterone. Metabolic profiling showed that the conversion of Gly to Ala in both positions of the I‐helix kink (G292A and G293A) reduced CYP21A2 activity to about 60% of the WT activity, whereas substituents with more sizeable side chains more profoundly inhibited the enzyme function. For instance, G292S/SNP and G293S mutations had less than 10% of the WT activity, and G292R/SNP, G292V, and G293V mutations had almost no activities at all (Figure 2A,D). CYP7A1 catalyzes the first and rate‐limiting step and synthesizes 7‐hydroxycholesterol in bile acid biosynthesis. Mutating Ala to a smaller Gly (A285G) had about 50% of the WT activity, but other mutations (A285R, A285V and A285T/SNP) led to the loss of function (Figure 2B,E). CYP7A1 has a Ser at the second position of the I‐helix kink. Mutating it into residues with smaller or equivalent side chains (e.g. S286G, S286A, S286V and S286L/SNP) had almost negligible impacts on CYP7A1 activity (Figure 2B,E). Unlike CYP21A2 and CYP7A1, which catalyze endogenous metabolites, CYP2A6 mainly oxidizes xenobiotics. CYP2A6 is involved in nicotine metabolism and implicated in nicotine resistance and tobacco addiction. Interestingly, G301A/SNP had no impact on the biosynthesis of 7‐hydroxycoumarin, whereas the G302A/SNP mutation reduced the activity to ∼40% of the WT activity (Figure 2C,F). Other mutations (G301S, G301V/SNP, G302S, G302V and G302D/SNP) abrogated the enzyme function (Figure 2C,F). In total, even though only three human CYPs were examined, we have already characterized eight non‐synonymous SNPs, six of which have not been characterized before. It is almost certain that other uncharacterized SNPs, particularly those substitutions with more sizeable side chains, might also disrupt the enzyme activity and be implicated in metabolic disorders and abnormal drug metabolism.

**FIGURE 2:**
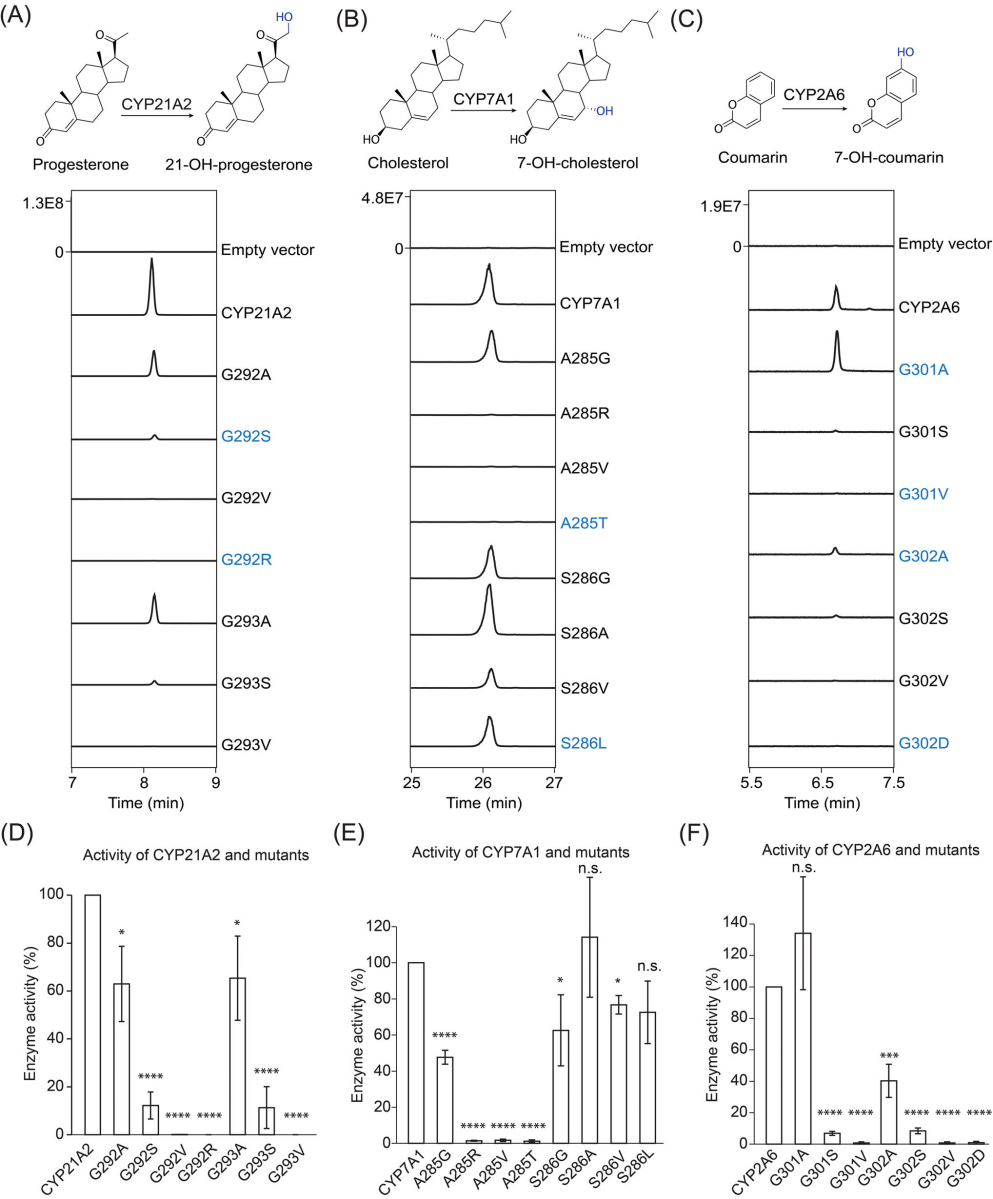
Functional characterization of single‐nucleotide polymorphisms (SNPs) of the I‐helix kink in different Cytochrome P450s (CYPs). (A–C) The enzyme activities of the wild‐type (WT) and mutants of CYP21A2 (A), CYP7A1 (B), and CYP2A6 (C) were revealed by liquid chromatography‐tandem mass spectrometry (LC‐MS/MS). SNPs for each CYP enzyme are shown in blue. (D–F) The quantitative analyses of enzyme activities of WT and mutants of CYP21A2 (D), CYP7A1 (E), and CYP2A6 (F). The activity of WT was set to 100 %. The statistical analyses between WT and mutants were performed by using a two‐sample *t‐*test assuming equal variance. * *p*‐value < .05, ** *p*‐value < .01, *** *p*‐value < .001, and **** *p*‐value < .0001.

To gain mechanistic insights into how the mutations of the I‐helix kink may affect CYP catalysis, we used the crystal structure of the CYP2A6‐coumarin complex as an example (PDB: 1Z10) and performed molecular dynamics (MD) simulations. Typically, the CYP‐mediated oxidation installs one oxygen atom from the heme iron‐oxygen intermediate into the substrate, which requires the participation of electrons from NAD(P)H and protons delivered by water molecules via the hydrogen‐bond network inside the active site.^13^, ^14^, ^15^ A solvent channel, formed among the E, F, and I helices, is important for the entry and egress of water molecules and is implicated in proton delivery.^16^ The MD simulations enabled us to examine how the solvent channel formation, proton delivery, and the distance between the oxidation site and the catalytic center might be affected by the mutations of the kink during catalysis. Only subtle fluctuations were observed in loop C‐D and H‐I over 400 ns of simulations for WT and the mutants (Figure S2). Because previous studies suggest that E304 is important to the opening of the solvent channel in CYP2A6 through its interaction with R311 during MD simulations,^16^, ^17^ we measured the average distance between the side chain of E304 and R311 in WT and all mutants. Apart from G301A, a significantly longer distance was observed between E304 and R311 for all other mutants (Figure 3A), which causes the reduced interactions of these two residues (Figure 3B). This might disrupt water access and compromise the enzyme activity (Videos S1 and S2). We also measured the distance between the oxygenation site of coumarin (C7) and the iron atom of heme (Fe). Only the G301V mutant was affected (Figure 3C,D); other mutants remained largely unchanged (Figure S3A,B). The larger side chain at the position of 301 might create steric hindrance that prevents the proper positioning of the substrate inside the active site (Figure S3C). On the other hand, the larger side chain at the position of 302 affected the hydrogen bond formed between the side chain of T305 and the carbonyl oxygen of G301 (G301_CO…HO_T305) (Figure 3E), which is implicated in the proton delivery during catalysis,^6^, ^8^, ^18^ and might compromise the geometry of the heme iron (Figure 3F). All those unfavourable changes were not observed in the G301A mutant (Figure S3), consistent with the unaffected activity.

**FIGURE 3:**
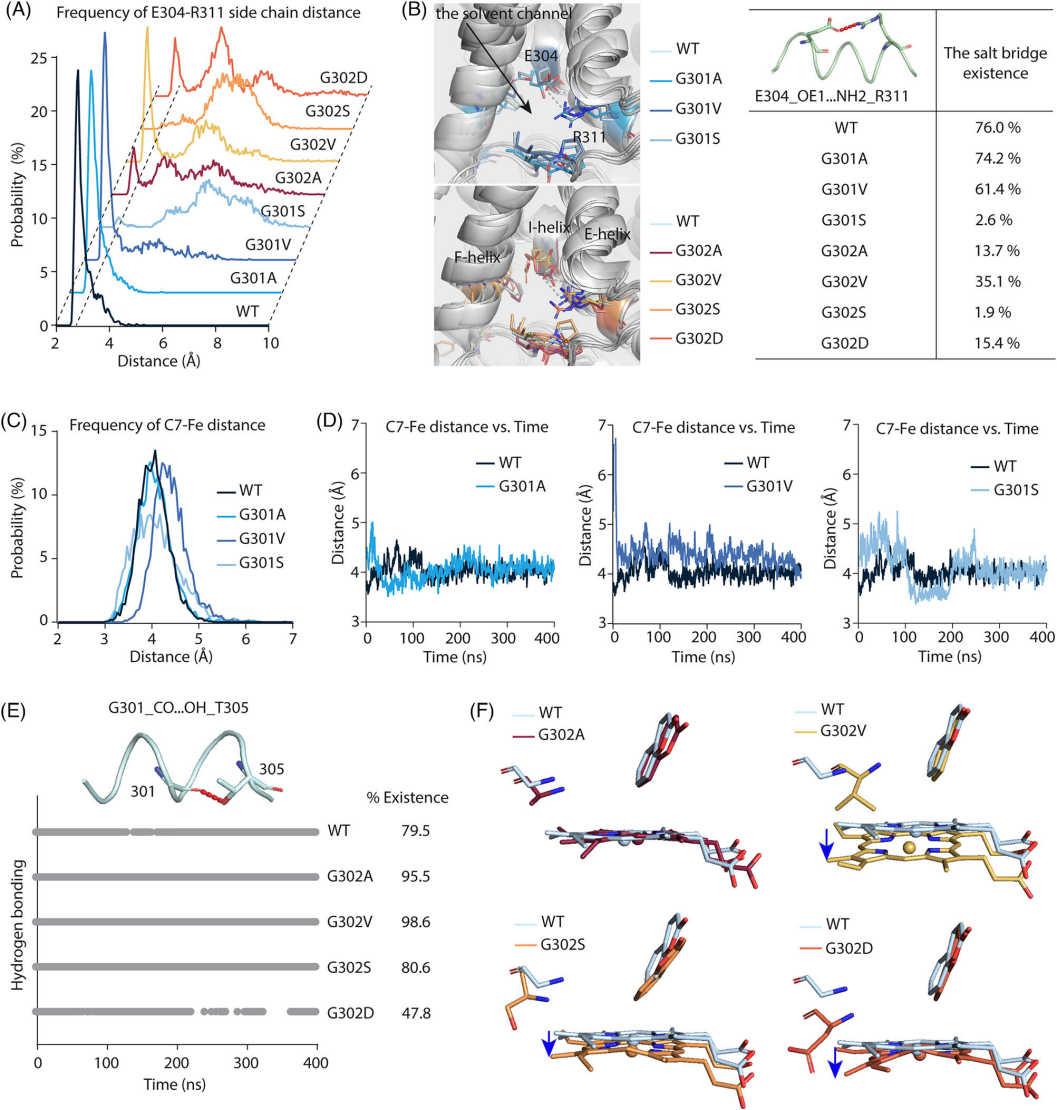
The structural changes of the solvent channel, the hydrogen bonding networks, the substrate binding pocket, and the heme geometry between the wild‐type (WT) and mutants of CYP2A6 during 400‐ns simulations. (A) The probability distributions of the distance between the side chain of E304 and R311 for WT CYP2A6 and all mutants. All line graphs share the same x‐ and y‐axis limits. The line graphs for mutants are shifted parallelly from WT. (B) The structural changes of the solvent channel for CYP2A6 WT and all seven mutants and the corresponding occupancy of the salt bridges between E304‐R311. (C) The probability distributions of the C7‐Fe distance for WT and G301 mutants over 400‐ns simulations. (D) The running average of the C7‐Fe distance vs. MD time for WT and G301 mutants, respectively. The running average of the C7‐Fe distance of each sample was plotted in R using ggplot2, with 11 frames used for the calculation. (E) The hydrogen bonding of G301_CO…HO_T305 versus time graph and the occupancy of the hydrogen bond for WT and G302 mutants. (F) The structural changes of the heme group in G302 mutants by using gmx cluster analyses. The cluster 1 output (with the least RMSD value) of each sample was used for the structure comparison. The blue arrows represent the shift of heme between WT and mutants.

Because of the importance of CYPs in drug metabolism, our findings might have immediate implications for precision medicine. The human CYP3A4 plays an important role in the newly developed paxlovid treatment (consisting of nirmatrelvir and ritonavir) for coronavirus disease 2019 (COVID‐19). Nirmatrelvir acts against severe acute respiratory syndrome coronavirus 2 protease, whereas ritonavir inhibits CYP3A4 activity to maintain nirmatrelvir concentration within the therapeutic range.^19^ As both compounds are substrates of CYP3A4, their activity must be considered when paxlovid is prescribed. An A305S mutant of CYP3A4 (equivalent to G301 in CYP2A6) was found in the dbSNP database. We performed 400‐ns simulations of the WT and A305S mutant using a CYP3A4‐midazolam complex structure as the model (PDB: 5TE8). The Cα‐based RMSF values of the WT and A305S mutant revealed the motions of the F/G region, and G‐H and H‐I loops, which was in line with the reported flexible feature of these regions (Figure S4).^20^, ^21^, ^22^, ^23^ Consistent with what we found in CYP2A6, the A305S mutant caused a spatial hindrance at the binding pocket (Figure 4A,B), suggesting compromised enzyme activity. To test this, we assayed the activity of CYP3A4 and A305S mutants against midazolam. The activity of the A305S mutant was largely compromised as predicted (Figure 4C,D). Consistently, we found that the A305S mutation compromised the metabolism of nirmatrelvir (Figure 4E,F). Since our results suggested that the A305S mutation compromises the metabolism of paxlovid, treating COVID‐19 patients carrying this mutation should be more cautious.

**FIGURE 4:**
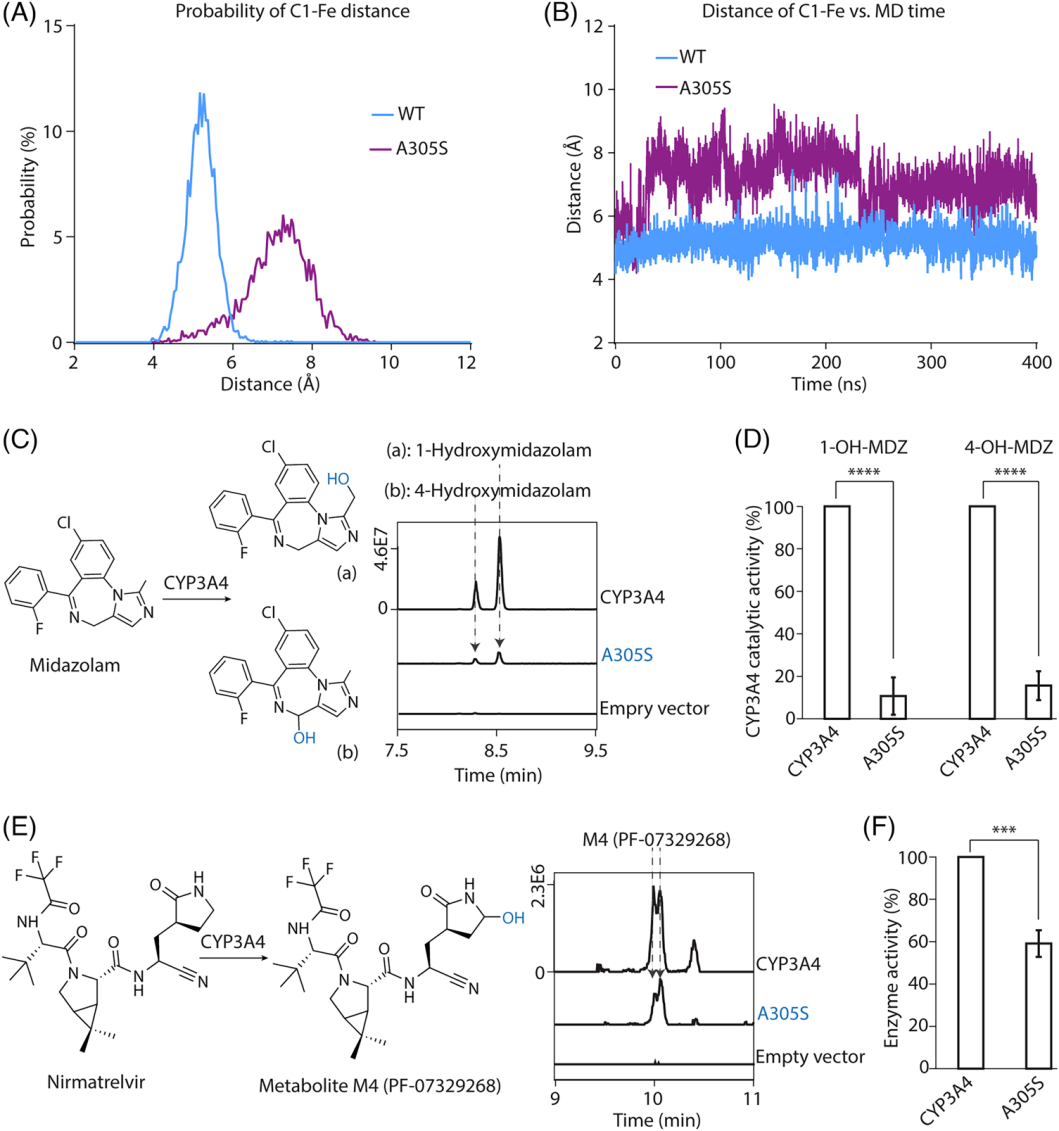
Molecular dynamics (MD) analyses of CYP3A4 wild type and A305S mutant and their catalytic activities. (A) The probability distribution of the C1‐Fe distance for wild‐type (WT) and A305S mutant over 400‐ns simulations. (B) The distance of C1‐Fe versus time graph for WT (blue) and A305S (purple) mutant during 400‐ns simulations. (C) The liquid chromatography‐tandem mass spectrometry (LC‐MS/MS) analyses of 1‐hydroxymidazolam and 4‐hydroxymidazolam produced by CYP3A4 and A305S mutant. (D) The statistical analyses of the enzyme activities against midazolam. * *p*‐value < .05, ** *p*‐value < .01, *** *p*‐value < .001, and **** *p*‐value < .0001. (E) The LC‐MS/MS analyses of nirmatrelvir metabolite M4 (PF‐07329268) produced by WT and A305S mutant. (F) The statistical analyses of the enzyme activities against nirmatrelvir . * *p*‐value < .05, ** *p*‐value < .01, *** *p*‐value < .001, and **** *p*‐value < .0001.

Taken together, we revealed that the I‐helix kink plays an “outsized” role such that subtle changes might lead to detrimental consequences in functionally unrelated human CYPs with low sequence similarities. Since there are many SNPs in this region in humans, we will not be surprised that those SNPs with more sizeable side chains might also disrupt the enzyme activity and be implicated in metabolic disorders and abnormal drug metabolism, and therefore, should be considered for precision medicine. On the other hand, the structural flexibility of the CYPs must be considered, and the impact of each SNP should be experimentally tested before drawing any conclusion.

## Supporting information

Supporting Information

Supporting Information

Supporting Information

Supporting Information

Supporting Information
