# Supplementary material for: The “outsized” role of the I‐helix kink in human Cytochrome P450s
Source: Clin Transl Med. 2023 Sep 15;13(9):e1378. doi: 10.1002/ctm2.1378 (PMC10502461; doi:10.1002/ctm2.1378)
Supplement: Supplementary file 5 — Supporting Information [file CTM2-13-e1378-s003.docx]

Supplementary Materials for

**The “outsized” role of the I-helix kink in human Cytochrome P450s**

Jingjing Zhang^1,2#^, Fengting Liu^1#^, Yaran Suo^3^, Dudu Tong^1^, Jinyu Hu^1^, Hai-Ning Lyu^3^, Jingjing Liao^3^, Jiaqi Wang^4*^, Jigang Wang^1,3*^, Chengchao Xu^1,3,5*^

^1^Department of Nephrology, Shenzhen Key Laboratory of Kidney Diseases, Shenzhen Clinical Research Centre for Geriatrics, Shenzhen People's Hospital, The First Affiliated Hospital, Southern University of Science and Technology, Shenzhen 518020, China

^2^Integrated Chinese and Western Medicine Postdoctoral Research Station, Jinan University, Guangzhou 510632, China

^3^State Key Laboratory for Quality Ensurance and Sustainable Use of Dao-di Herbs, Artemisinin Research Center, and Institute of Chinese Materia Medica, China Academy of Chinese Medical Sciences, Beijing, 100700, China

^4^School of Pharmaceutical Sciences (Shenzhen), Shenzhen Campus of Sun Yat-sen University, Shenzhen 518107, China

^5^College of Integrative Medicine, Laboratory of Pathophysiology, Key Laboratory of Integrative Medicine on Chronic Diseases, Fujian University of Traditional Chinese Medicine, Fuzhou, 350122, Fujian, China

^#^These authors contributed equally to the study.

*Correspondence:

[ccxu@icmm.ac.cn](mailto:ccxu@icmm.ac.cn) (C.X.);

[jgwang@icmm.ac.cn](mailto:jgwang@icmm.ac.cn) (J.W.)

[wangjq59@mail.sysu.edu.cn](mailto:wangjq59@mail.sysu.edu.cn) (J.W.)

**This PDF file includes:**

Materials and Methods

Supplementary Figure S1 to S6

Supplementary Table S1 to S2

Supplementary Video S1 to S2**Materials and Methods**

### Phylogenetic analyses

The phylogenetic tree with 1000 bootstrap was built by MEGA11 ^1^ using the maximum-likelihood method. The whole amino acid sequences of 56 human CYPs are used for phylogenetic tree. Bootstrap values are indicated at the tree nodes.

### Construction of plasmids and site-directed mutagenesis of human CYPs

The full-length cDNA of human CYP2A6 (CCDS12568.1), CYP21A2 (CCDS4735.1), CYP7A1 (CCDS6171.1), and CYP3A4 (CCDS5674.1) with a C-terminal FLAG tag were constructed into the expression vector pcDNA3.1+, respectively. Mutations for each CYP gene were introduced using the Dpn1-mediated site-directed mutagenesis ^2^. In brief, the primers were designed to include single mutations (**Table S2**) and the mutation was introduced by PCR. The PCR products were treated with Dpn1 enzyme (New England Biolabs) for 1 h at 37°C to digest the parental DNA and the resulting plasmids were transformed into DH5α competent *E. coli* cells. Correct mutations were verified by DNA sequencing. Finally, the correct plasmid was extracted using the Plasmid Miniprep kit (Tiangen biotech (Beijing) CO., LTD.).

### Expression of wild type and mutants of human CYPs in 293T/17 cells

The HEK 293T/17 human kidney cells (Procell) were used for transient transfection. A density of 4 x 10^4^ cells was seeded per well in 24-well plates and recovered overnight in Dulbecco’s Modified Eagle’s Medium (Gibco) with 10% fetal bovine serum (Hyclone), 100 U/mL penicillin and 100 µg/mL streptomycin. Then the cells were transfected with 0.5 µg of plasmids carrying either WT or mutant CYPs and cultured at 37°C under a humidified atmosphere of 5% CO_2_ for 48h for CYP expression. The empty vector was used as negative control.

### Immunoblotting analysis

Immunoblotting analysis was used to visualize the expression level of WT and mutant CYPs. Proteins were harvested directly from plated cells after 48 h of transfection. A 10 µg of total proteins from each sample was separated on a 10% SDS-polyacrylamide gel electrophoresis (SDS-PAGE), transferred to a polyvinylidene fluoride film, and blocked by 5% milk. Immunoblot detection was performed, using the anti-FLAG antibody purchased from Sigma-Aldrich (all the CYPs contained the FLAG tag) and the glyceraldehyde 3-phosphate dehydrogenase antibody (anti-GADPH, Proteintech). After incubation with goat HRP-conjugated secondary antibodies (Thermo Fisher Scientific), the blots were incubated with the ECL solution (Bio-Rad) and the chemiluminescent signals were detected using the ChemiDoc Touch Imaging system (Bio-Rad).

### Enzymatic assays for wild type and mutants of human CYPs

The HEK 293T/17 cells carrying either WT or mutants of different human CYPs were fed with 0.5 µM of the corresponding substrate (*i.e.* progesterone (Tocris bioscience) for CYP21A2; coumarin (Shanghai Yuanye Bio-Technology Co., Ltd) for CYP2A6; midazolam (Sigma) for CYP3A4; cell intracellular cholesterol for CYP7A1) and 0.4 mM of the β-NADPH cofactor (Aladdin) and incubated at 37°C for 30 min. Then, each sample containing culture medium and cells was mixed with chloroform and methanol (v/v, 500 uL : 300 uL) vigorously and centrifuged at 12000 rpm for 10 min. The chloroform layer was carefully transferred into a new 1.5 mL eppendorf tube and dried in the SpeedVac vacuum concentrator. The chloroform extraction was purified again with 500 µL of chloroform, dried in the vacuum concentration, and was stored at − 20 °C until analysis. Each experiment was performed in three replicates.

### LC-MS/MS analysis

A Q Exactive Focus mass spectrometer (Thermo Scientific) coupled to an Ultimate 3000 rapid separation LC system (Thermo Scientific) was used for liquid chromatography-high resolution mass spectrometry (LC-MS) analysis in this study. Each sample was dissolved in 50 µL of 80% methanol, sonicated for 5 min, centrifuged at 13000 rpm for 10 min at 4 °C, and analyzed by LC-MS using the reverse-phase chromatography system consisted of a 100 mm C18 column (Hypersil GOLD VANQUISH C18 column, 1.9 µm, 100 x 2.1 mm, Thermo Fisher Science).

For LC-MS analysis of less non-polar metabolites from CYP21A2, CYP2A6 and CYP3A4, 0.1% formic acid (FA) in water (solvent A) and 0.1% FA in acetonitrile (solvent B) were used. A gradient from 30% to 90% within 12 min for CYP21A2, while a gradient from 10% to 90% solvent B within 15 min for CYP2A6 and CYP3A4 were performed, respectively.

For LC-MS analysis of strong non-polar metabolites from CYP7A1, 0.5 mM of ammonium acetate in water (solvent A) and 0.5 mM ammonium acetate in methanol (solvent B) were used^3^, and a gradient from 65% to 99% solvent B within 25 min were performed. The flow rate was 0.2 mL/min and the injection was 2 µL. The mass spectrometer was operated in the positive mode, with a scan range of 100 ~ 1000 m/z and a resolute ion of 70,000 for both MS and MS/MS.

### Quantitative and statistical analyses of the enzyme activity

The enzyme activity was measured by the peak area of the product (**Figure S5, S6**). The activity of WT was set to 100%. The enzyme activity of each sample was calculated by the following equation: Enzyme activity = (the peak area of each sample/ the peak area of WT) × 100 %. The data was expressed as means ± S.D. (the standard deviation) of the three replicates. The statistical significance between WT and mutants was analyzed by using the two-sample *t-*test assuming equal variance. A *p-*value below 0.05 was regarded as statistically significant. * represents *p-*value < 0.05, ** represents *p-*value < 0.01, *** represents *p-*value < 0.001, and **** represents *p-*value < 0.0001.

### System set-up

To construct the initial structures, the structures of CYP2A6 in complex with coumarin (PDB: 1Z10) and CYP3A4 in complex with midazolam (PDB: 5TE8) were used. The preparation of topology files and molecular dynamic (MD) simulations were the same for both CYP2A6 and CYP3A4. Here, we used CYP2A6 as an example to describe the detailed processes. Only one CYP2A6-coumarin complex in PDB 1Z10 was kept, with water molecules removed. We then performed parameterizations for the system using the Metal Centre Parameter Builder program (MCPB.py) released in AmberTools21 (http://[ambermd.org/tutorials/advanced/tutorial20/mcpbpy_heme.php](https://ambermd.org/tutorials/advanced/tutorial20/mcpbpy_heme.php)) ^4^. Firstly, we extracted non-standard residues, including the heme group without the iron metal and the coumarin from the complex, and prepared their parameters. The heme group and coumarin were added hydrogens using reduce module then parameterized using the antechamber and the parmchk2 programs. Here, the charge of heme group was generated by the AM1-BCC charge method and the general AMBER force field (GAFF) was used for those non-standard residues. The heme was in a pentacoordinated Fe^III^ state. Secondly, we extracted the iron metal into an independent residue and parameterized it by using a python script (metalpdb2mol2.py). Then, we protonated the amino acids at pH 7.2 and 0.15 M of salinity using the webserver H++ (<http://biophysics.cs.vt.edu/H>++), which generated the topology and coordinate files for the protein ^5-7^. Since the iron metal coordinates to an axial cysteine sidechain (S), this cysteine should be negatively charged. Thus, we deleted the “HG” atom of the cysteine residue and renamed it to “CYM”. Finally, we combined all the above parameters of all standard and non-standard residues into a single PDB file using ambpdb program and renumbered the file using pdb4amber program. After generating the fingerprint files of the small, standard and large models of the metal cluster (the coordination of iron to four nitrogen atoms of heme group and the cysteine sidechain) by using the MCPB.py programs, we performed the quantum calculations for both the small and large models based on the B3LYP/6-31G* level of theory using the Gaussian 16 software ^8^. Here, the restraint electrostatic potential (RESP) method was used to generate the partial charges of atoms of heme moiety. Then we used the Seminario method to generate the force field parameters. The final amber topology and coordinate files were generated by using the tleap module in AmberTools21. The AMBER ff14SB force field was used for the protein and the whole system was immersed in an octahedral box of TIP3P water molecules and counter ions (Na^+^ and CL^-^). As a results, a total of 41679 water molecules were added to the solvent box and five CL- ions were supplemented to the simulation box to maintain the electric neutrality of the system. For mutations, we changed the corresponding tleap input files and then performed the tleap command. Finally, we transferred topology and coordinate files into GROMACS - recognized topology files by acpype program ^9^.

### Molecular dynamics simulations

The system was simulated using GROMACS (version 2021. 5) ^10^. The whole system was firstly minimized to remove any bad contact, then slowly heated from 0 to 300 K in a 100 ps of MD simulations using an isothermal-isochoric (NVT) ensemble, finally equilibrated with the isothermal-isobaric (NPT) in a 100 ps of simulations under constant temperature and pressure. The temperature and pressure were controlled by the Berendsen coupling algorithm with a time constant of 0.1 and 1.0 ps, respectively. The simulation for WT and mutants of CYP2A6 and CYP3A4 system were 400 ns and the integration time step was 2 fs. We then used the periodic boundary condition (PBC) for the MD trajectories. The structures used for analysis were sampled every 100 ps of the trajectories, thus, each complex consists of 4001 snapshots. Root-mean-square deviation (RMSD) and root-mean-square fluctuation (RMSF) values were computed by *gmx rms* and *gmx rmsf* programs of GROMACS services. All the hydrogen bonds analyses in this study were performed using the program *gmx hbond* and the distances between two atoms were calculated with *gmx distance* program. The running average of the distance data of each sample was plotted in R using ggplot2, with 11 frames used for the calculation. The CYP2A6-coumarin complex was performed with the *gmx cluster* tool using the GROMOS clustering algorithim ^11^, with Cα-based RMSD calculation and the cutoff of 0.13 nm. The output for each cluster is the structure with the least RMSD.

### The solvent channel analysis

The CAVER Analyst 2.0 BETA ^12^ was used for the solvent channel calculation. One hundred snapshots of each system were captured from 400 ns of MD simulations. The MD trajectories were loaded into CAVER Analyst 2.0 as the Gromacs format. The solvent channel was calculated surrounding residues from helix E, F, and I and β4. The minimum probe radius was set to 1.25 Å and other parameters were remained as defaults.

Supplementary figure legends

Supplementary Figure S1

The protein expression levels of the wild type and mutants of CYP21A2, CYP7A1, CYP2A6 and CYP3A4. Empty vector was used as control, and the human glyceraldehyde 3-phosphate dehydrogenase (GAPDH) served as a loading control for each experiment.


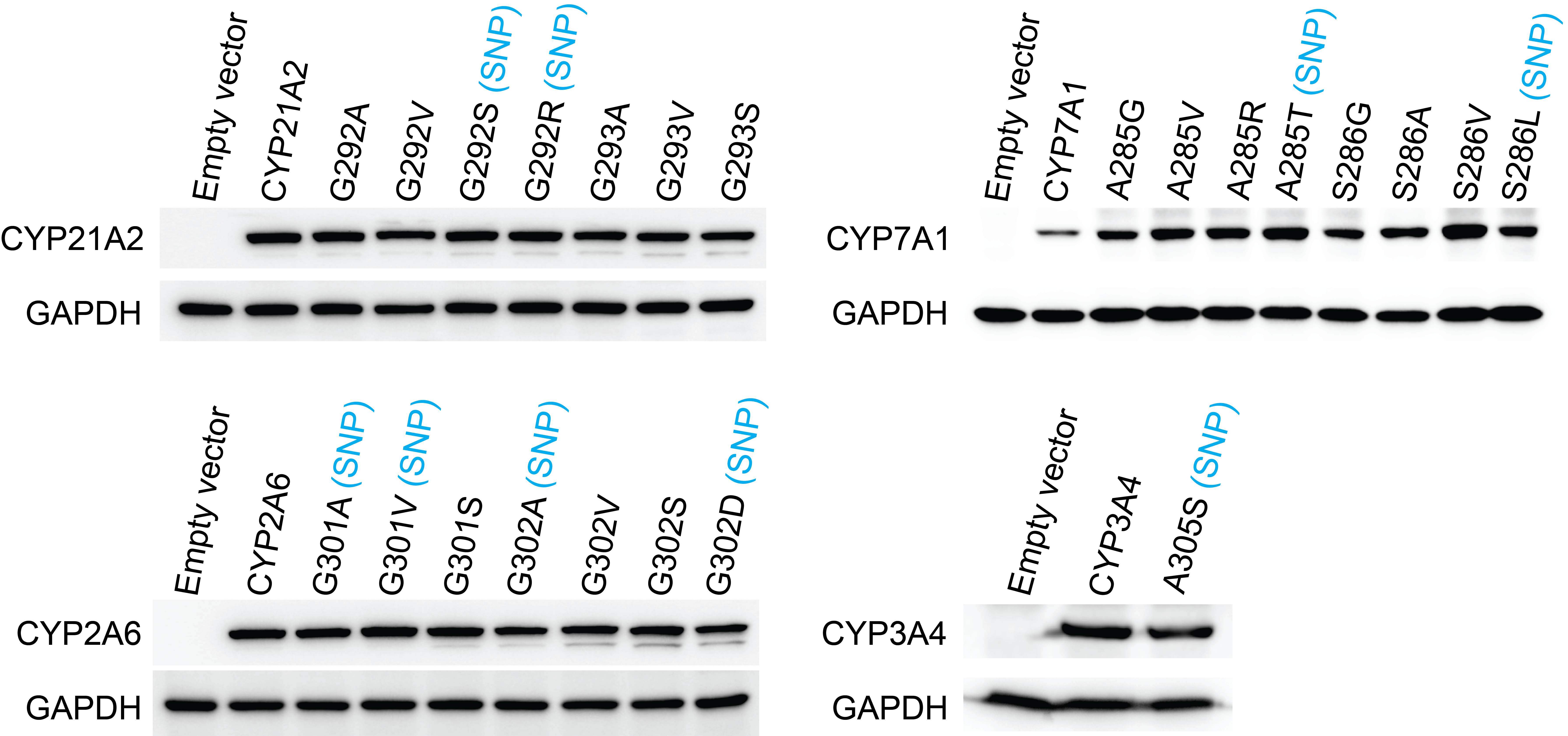


Supplementary Figure S2

The protein-based RMSD plots and the Cα-based RMSF values for CYP2A6 wild type and mutants. (A) The RMSDs of main chain atoms vs. time graph for CYP2A6 wild type and mutants during 400 ns of simulations. (B) The analysis of RMSF trajectories per backbone residue for CYP2A6 wild type and mutants in 400 ns of simulations. The RMSF vs. residue graph for each mutant (blue) was compared with that of wild type (purple). The subtle fluctuations were observed in loop C-D and H-I for WT and mutants.


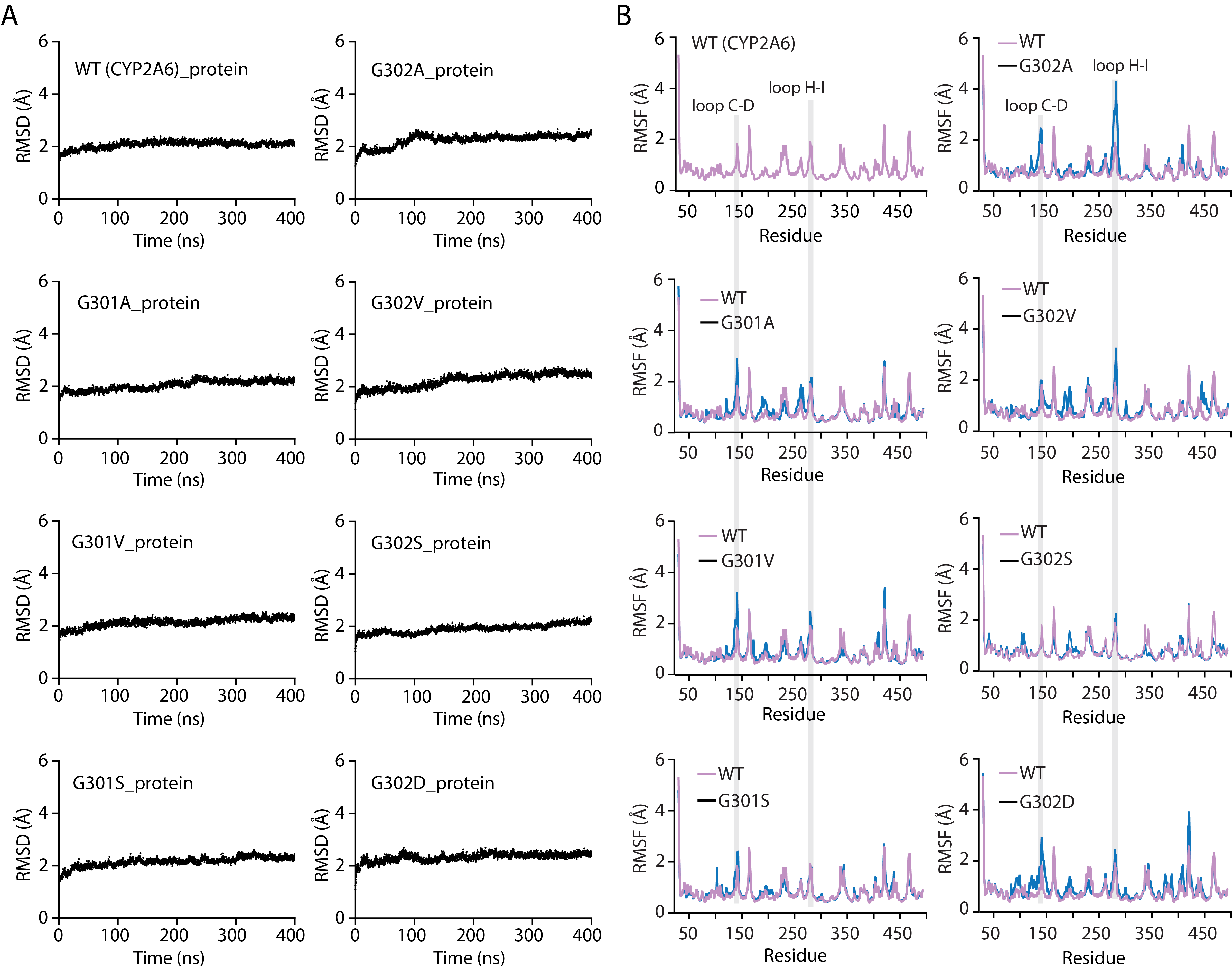


Supplementary Figure S3

The changes of the distance between C7-Fe for G302 mutants, the hydrogen bonding networks for G301 mutants, and the structural changes for all mutants during the 400-ns simulations. (A) The probability distributions of the distance between C7 atom of coumarin and the iron atom of heme for CYP2A6 WT and G302 mutants during 400 ns of simulations, respectively. (B) The running average of C7-Fe distance vs. MD time graph for WT and G302 mutants. The running average of each sample was plotted in R using ggplot2, with 11 frames used for the calculation. (C) The structural changes between WT and all mutants by comparing the cluster 1 output with the least RMSD of each sample. The cluster 1 of each sample was generated by using *gmx cluster*. The blue arrows represent the shift of coumarin between WT and mutants. (D) The hydrogen bond between the carbonyl oxygen of residue 301 (301aa_C=O) and the side chain hydroxyl of T305 (OH_T305) vs. time graph for WT and G301 mutants and the corresponding hydrogen bond occupancy analyses over the 400 ns of simulations. (E) The structural changes on heme geometry between WT and G301 mutants. The structures used for comparisons were the cluster 1 outputs of each sample by using *gmx cluster*. The blue arrows represent the shift of heme between WT and mutants.


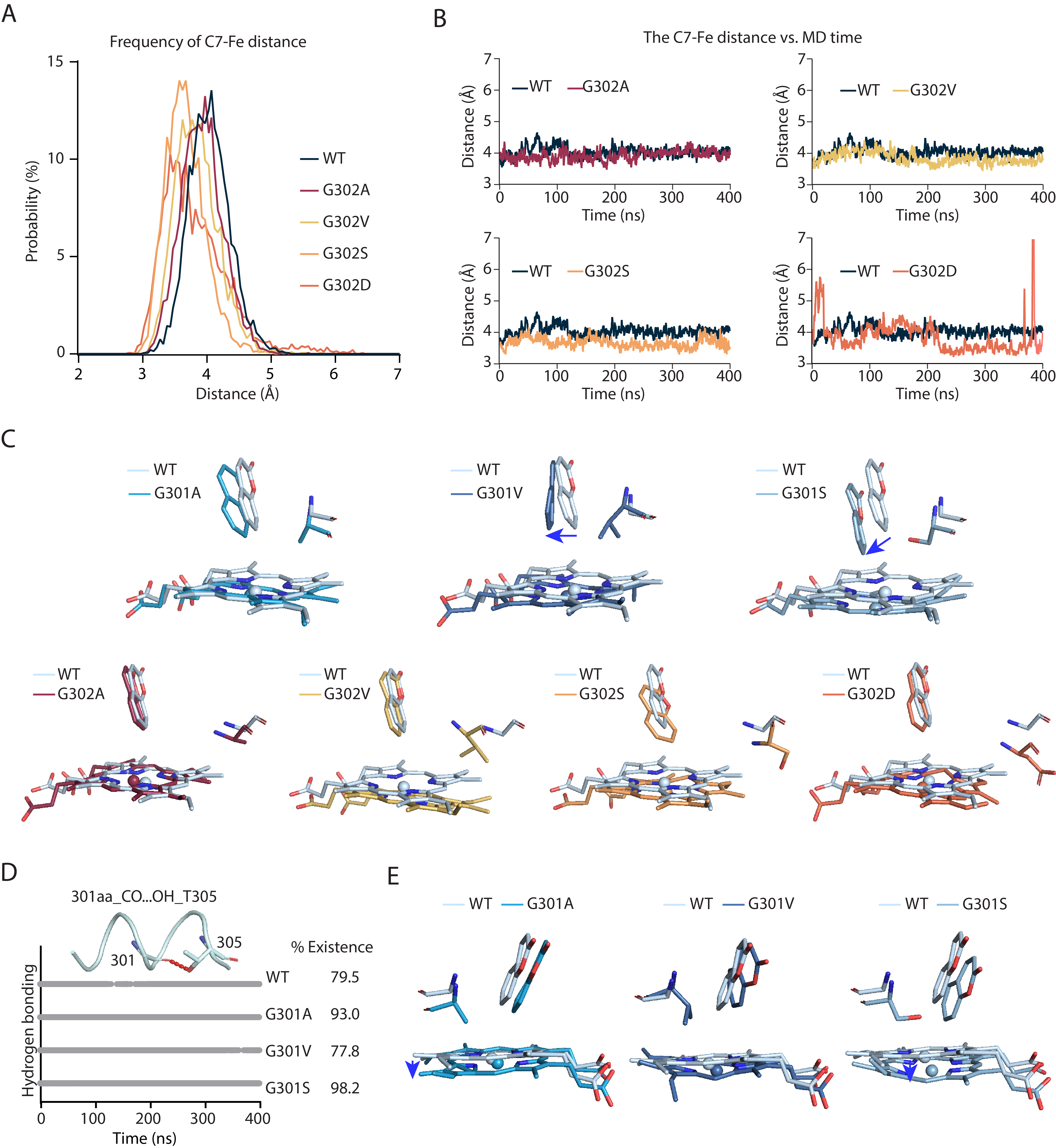


Supplementary Figure S4

The protein-based RMSDs and Cα-based RMSFs for the CYP3A4 wild type and A305S mutant. (A) The RMSD plots of the main chain atoms vs. time graph for the WT (blue) and A305S mutant (purple) during 400 ns of simulations. (B) The RMSF plots calculated based on the Cα residues over 400-ns simulations of the WT (blue) and A305S mutant (purple), respectively.


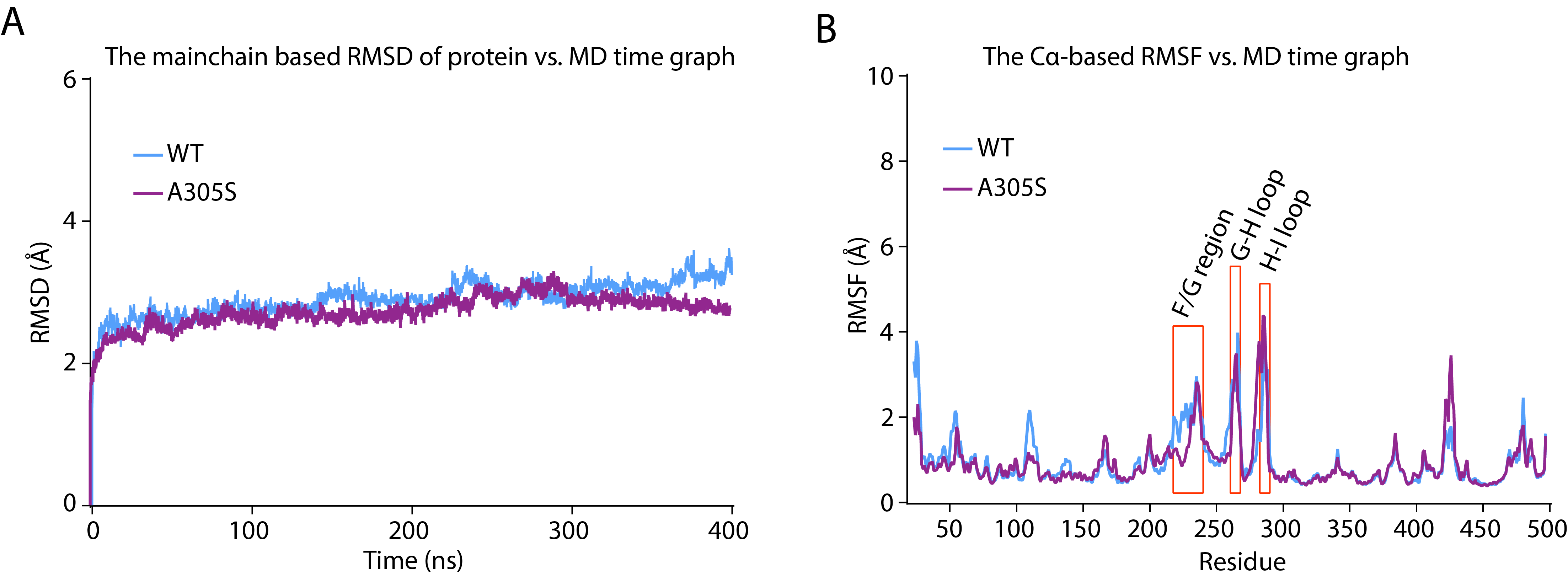


Supplementary Figure S5

The mass spectrometry evidence for 21-hydroxyprogesterone (21-OHP), 7α-hydroxycholesterol (7-OH cholesterol), 7-hydroxycoumarin (7-OH coumarin), 1-hydroxymidazolam (1-OH MDZ) and 4-hydroxymidazolam (4-OH MDZ), and nirmatrelvir metabolite M4 (PF-07329268). (A) The extracted ion chromatograms (EICs) of the analytical standards and the metabolites, related to CYP21A2 (A), CYP7A1 (B), CYP2A6 (C), and their corresponding MS/MS patterns. The inserts were the mass spectra at [M+H]^+^. (D) EICs and the corresponding MS2 spectra for CYP3A4 metabolites, 1-OH MDZ and 4-OH MDZ. Here, the 1-OH and 4-OH MDZ were identified by their reported MS/MS ions ^13-15^. (E) EICs of *m/z* 498.23 and *m/z* 516.24 and the MS1 and MS2 spectra for metabolite M4. Here, the M4 was identified by the reported MS/MS ions ^16^.


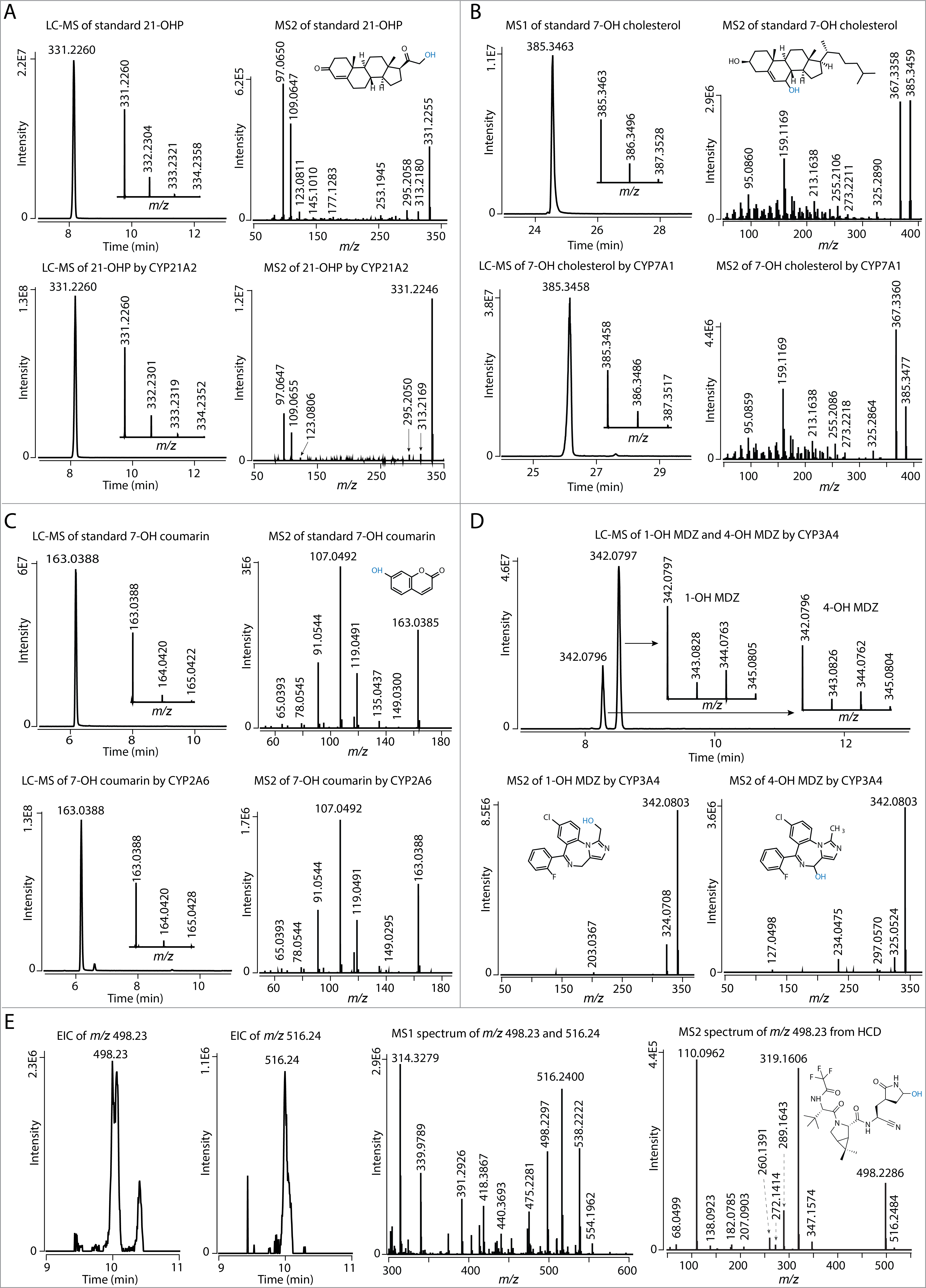


Supplementary Figure S6

The LC-MS chromatography analyses of the corresponding products from wild type and mutants of CYP21A2 (A), CYP7A1 (B), CYP2A6 (C), and CYP3A4 (D-E) in three replicates.


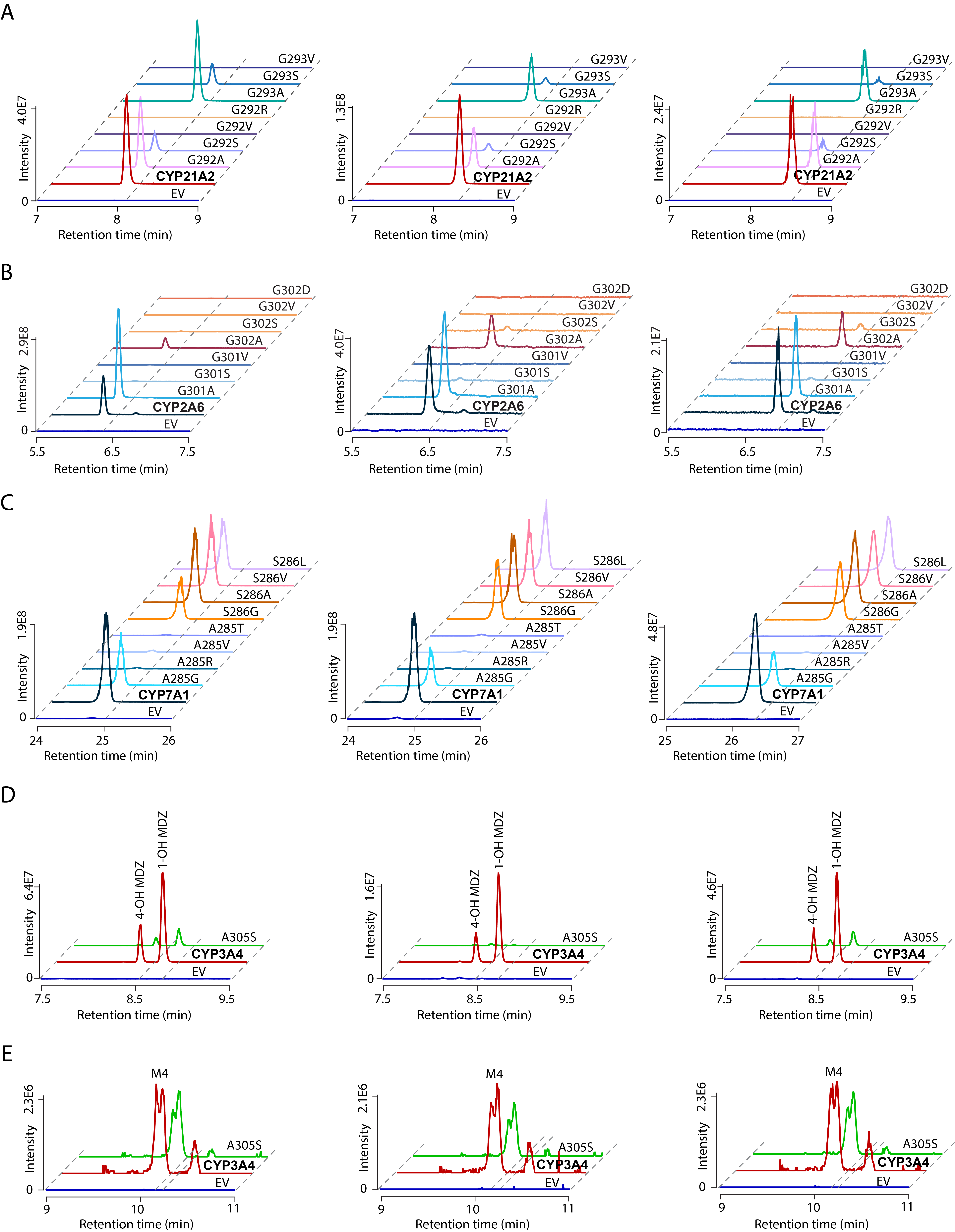


Supplementary Table S1

The list of the non-synonymous polymorphisms of the I-helix kink in human CYPs.

Supplementary Table S2

The list of primers and plasmids used in this study.

Supplementary video S1

Movie of MD trajectory showing the state of the solvent channel for wild type CYP2A6-coumarin complex during 400 ns of simulations. The solvent channel was shown in blue. The residues E304 and R311 were colored in pink, and heme and coumarin were colored in light grey.

Supplementary video S2

Movie of MD trajectory showing the state of the solvent channel for the mutant G302D-coumarin complex during 400 ns of simulations. The solvent channel was shown in blue. The residues E304 and R311 were colored in red, and heme and coumarin were colored in light grey.

**Supplementary References**

1. Tamura K, Stecher G, Kumar S. MEGA11: Molecular Evolutionary Genetics Analysis Version 11. *Mol Biol Evol.* 2021;38(7):3022-3027.

2. Liu H, Naismith JH. An efficient one-step site-directed deletion, insertion, single and multiple-site plasmid mutagenesis protocol. *BMC Biotechnol.* 2008;8(1):91.

3. Christ B, Xu C, Xu M, et al. Repeated evolution of cytochrome P450-mediated spiroketal steroid biosynthesis in plants. *Nat Commun.* 2019;10(1):1-11.

4. Li P, Merz KM, Jr. MCPB.py: A Python Based Metal Center Parameter Builder. *J Chem Inf Model.* 2016;56(4):599-604.

5. Anandakrishnan R, Aguilar B, Onufriev AV. H++ 3.0: automating pK prediction and the preparation of biomolecular structures for atomistic molecular modeling and simulations. *Nucleic Acids Res.* 2012;40(Web Server issue):W537-541.

6. Myers J, Grothaus G, Narayanan S, Onufriev A. A simple clustering algorithm can be accurate enough for use in calculations of pKs in macromolecules. *Proteins.* 2006;63(4):928-938.

7. Gordon JC, Myers JB, Folta T, Shoja V, Heath LS, Onufriev A. H++: a server for estimating pKas and adding missing hydrogens to macromolecules. *Nucleic Acids Res.* 2005;33(Web Server issue):W368-371.

8. Frisch Me, Trucks G, Schlegel HB, et al. Gaussian 16. In: Gaussian, Inc. Wallingford, CT; 2016.

9. Sousa da Silva AW, Vranken WF. ACPYPE - AnteChamber PYthon Parser interfacE. *BMC Res Notes.* 2012;5(1):367.

10. Abraham MJ, Murtola T, Schulz R, et al. GROMACS: High performance molecular simulations through multi-level parallelism from laptops to supercomputers. *SoftwareX.* 2015;1:19-25.

11. Daura X, Gademann K, Jaun B, Seebach D, Van Gunsteren WF, Mark AE. Peptide folding: when simulation meets experiment. *Angew Chem Int Ed Engl.* 1999;38(1‐2):236-240.

12. Jurcik A, Bednar D, Byska J, et al. CAVER Analyst 2.0: analysis and visualization of channels and tunnels in protein structures and molecular dynamics trajectories. *Bioinformatics.* 2018;34(20):3586-3588.

13. de Loor H, de Jonge H, Verbeke K, Vanrenterghem Y, Kuypers DR. A highly sensitive liquid chromatography tandem mass spectrometry method for simultaneous quantification of midazolam, 1′‐hydroxymidazolam and 4‐hydroxymidazolam in human plasma. *Biomed Chromatogr.* 2011;25(10):1091-1098.

14. Mooiman K, Maas‐Bakker R, Rosing H, Beijnen J, Schellens J, Meijerman I. Development and validation of a LC‐MS/MS method for the in vitro analysis of 1‐hydroxymidazolam in human liver microsomes: application for determining CYP3A4 inhibition in complex matrix mixtures. *Biomed Chromatogr.* 2013;27(9):1107-1116.

15. Dostalek M, Macwan JS, Chitnis SD, Ionita IA, Akhlaghi F. Development and validation of a rapid and sensitive assay for simultaneous quantification of midazolam, 1′-hydroxymidazolam, and 4-hydroxymidazolam by liquid chromatography coupled to tandem mass-spectrometry. *J Chromatogr B Analyt Technol Biomed Life Sci.* 2010;878(19):1629-1633.

16. Eng H, Dantonio AL, Kadar EP, et al. Disposition of Nirmatrelvir, an Orally Bioavailable Inhibitor of SARS-CoV-2 3C-Like Protease, across Animals and Humans. *Drug Metab Dispos.* 2022;50(5):576-590.
